# Automated Shear Strength Characterization at Micro Scales Based on a Microrobotic System

**DOI:** 10.3390/mi16101180

**Published:** 2025-10-19

**Authors:** Panbing Wang, Xintao Li, Xinyu Liu

**Affiliations:** 1 School of Instrument Science and Engineering, Southeast University, Nanjing 210096, China; 2 State Key Laboratory of Digital Medical Engineering, Nanjing 211189, China; 3 Jiangsu Key Lab of Robot Sensing and Control, Nanjing 210096, China

**Keywords:** micromanipulation, microrobot, soft sensors, mechanical property, shear strength characterization

## Abstract

Mechanical properties are critical for characterizing and fabricating advanced materials. While current characterization methods are well-established for the nanoscale and larger millimeter-scale, a significant gap exists in automated testing at the microscale. To address this, we propose an automated, rapid characterization method based on a microrobotic system. We first develop a 6-degree-of-freedom (DOF) microrobotic system for sample alignment and testing. An image processing method is then designed for real-time sample recognition, supplying essential feedback for both alignment and testing procedures. Furthermore, a soft force sensor is fabricated and calibrated to ensure precise force measurement. Experiments on copper wires and graphite films demonstrate the method’s high precision and reliability. This work provides a robust solution for microscale mechanical property characterization, offering significant potential for advanced material development.

## 1. Introduction

Mechanical property characterization is fundamental to understanding deformation behavior and critical for the development of advanced materials, including two-dimensional materials, nanowires, and thin films [1,2]. Among these properties, shear strength is a key parameter governing performance in applications from composite interfaces to flexible electronics [3]. Conventional methods for assessing shear strength at the nanoscale predominantly rely on advanced instrumentation such as Atomic Force Microscopy (AFM) [4], Transmission Electron Microscopy (TEM) [5], and Scanning Electron Microscopy (SEM) [6].

AFM-based techniques, such as Friction Force Microscopy (FFM) [7], Bimodal AFM [4], and nanoindentation, enable high-resolution mechanical characterization with nanometer precision [8,9]. These methods can be automated to varying degrees through software-controlled loading cycles [10,11,12], automated data acquisition [13], and real-time feedback systems [14]. Similarly, in situ TEM and SEM setups integrated with nanoindenters or MEMS devices allow for the direct visualization of deformation mechanisms during mechanical loading. For instance, Zhang et al. combined TEM with FIB technology and implemented software to track markers across sequential images to measure indentation depth [15]. Sato et al. utilized TEM and MEMS technologies to simultaneously measure deformation, force, and cross-sectional area during shear testing of a single asperity [5]. While the achievements mentioned above are primarily conducted at the nanoscale, the mechanical properties of materials at the micrometer scale are equally critical. For example, Dong et al. developed an automated torsion testing system for metallic glass wires [16,17]. However, for specific properties like shear strength, many methods remain manual, such as the use of an Interfacial Shear Strength (IFSS) instrument for testing carbon fibers [18].

Consequently, a significant gap exists in the automated characterization of shear strength for microscale materials (approximately 1–100 μm), precisely the domain of many functional materials like carbon fibers, metallic wires, and polymer films [19]. While nanoscale techniques (e.g., AFM) for handling microscale samples face challenges with sample interaction and measurement range [20,21], macroscale methods lack the necessary resolution and require complex preparation [22]. A further critical limitation is the reliance on manual processes for alignment, testing, and data analysis, which severely compromises throughput and repeatability [19,23]. This demands a need for a dedicated, automated platform to enable rapid and reliable shear testing of microscale specimens.

The microrobot is a promising technology for micro- and nanoscale manipulation, capable of nanometer-precision positioning and trajectory tracking [24]. They can be equipped with various end-effectors (e.g., grippers, needles) for diverse tasks and integrated with microscopes to provide in situ, high-resolution sample observation [25,26]. Consequently, microrobotic systems have been successfully applied in micro-nanoscale mechanical characterization [27]. However, previous microrobotic systems without full automation typically automated only part of the experimental procedure, such as loading cycles [11], data acquisition [13], or visualization of deformation mechanisms [15]. In contrast, a fully automated system enables automation of the entire mechanical characterization process. This distinction is particularly critical for microscale materials, where accurate alignment is a major challenge. For example, in the study of Feng et al. [18], the fiber and shear tool had to be manually aligned using a micromanipulation stage. Similarly, Barrios et al. [23] emphasized that fragile thin-film specimens required extremely cautious manual handling to ensure correct placement. Hence, a fully automated mechanical characterization process using a micro-robotic system, including automatic alignment, measurement, and data analysis has not yet been achieved.

To address these challenges, this paper presents an automated micro-robotic system for rapid mechanical characterization at the microscale. The framework of this paper is structured as follows. First, a 6-DOF microrobotic system is developed to enable precise sample alignment and application of shear force. Next, a machine vision algorithm is introduced to automate sample identification and facilitate closed-loop robotic alignment. Subsequently, a flexible Polyvinylidene Fluoride (PVDF) force sensor is designed and calibrated to allow for the real-time, in-situ measurement of shear forces. Finally, the effectiveness of the overall system is validated through automated shear strength tests performed on representative microscale materials. Experimental results demonstrate the high precision and repeatability of the proposed approach, as well as its adaptability to diverse sample geometries and dimensions. This system provides an efficient solution for rapid automated characterization, offering a practical platform for statistical analysis of material properties at the micro scale.

## 2. Microrobotic Manipulation System for Mechanical Characterization

### 2.1. Design of the Microrobotic System

**Figure 1:**
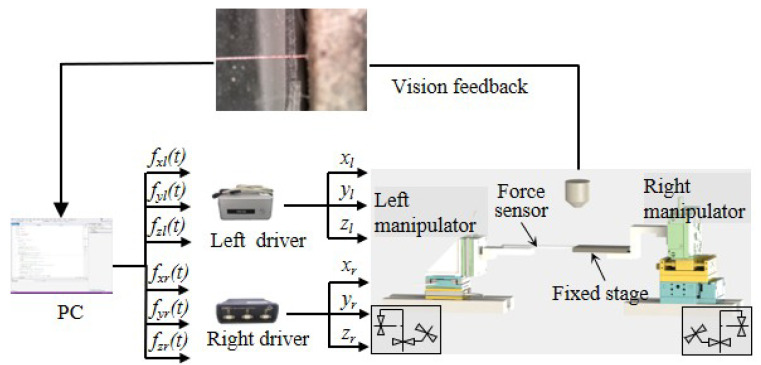
The architecture of the microrobotic system.

The architecture of the system for mechanical characterization is displayed in Figure 1. The microrobotic system consists of two manipulators. The left manipulator (ECS3030, Attocube, Germany) is an x–y–z stage with linear movement accuracy of 1 nm and a travel range of 20 mm. The right manipulator (MN3-18, Mechonics, Germany) is an x–y–z stage with a resolution of 10 nm and a travel range of 18 mm. The left manipulator assembles a soft force sensor for applying shear force on the sample, and the right manipulator fixes a steel cube for fixing the sample.

### 2.2. Coordinate System

**Figure 2:**
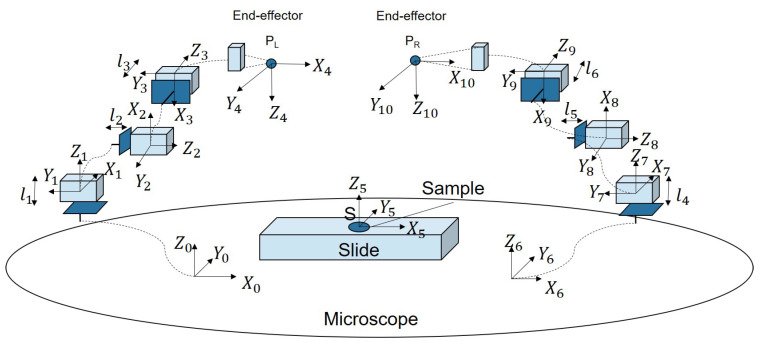
The coordinate system of the microrobotic system.

Before controlling the manipulator to the desired position, it is necessary to determine the position of its end-effector relative to the microscope’s field of view. To achieve this, a coordinate system is established, as illustrated in Figure 2. The base coordinate system {0} is fixed on the microscope stage, while the coordinate system of the left end-effector is defined as {4}. The transformation matrix between {0} and {4} is then derived as follows.

(1) T04=100Pxl0−10Pyl00−1Pzl0001

where the $P_{xl}$,$P_{yl}$, and $P_{zl}$ is the position of the left end-effector:

(2) $$\left[\begin{array}{c} P_{xl}\\ P_{yl}\\ P_{zl} \end{array}\right]=\left[\begin{array}{c} x_{01}-y_{12}+z_{23}-y_{34}+l_{2}\\ y_{01}+x_{12}-y_{23}+z_{34}+l_{3}\\ z_{01}+z_{12}+x_{23}-x_{34}+l_{1} \end{array}\right]$$

Similarly, the position of the right end-effector is derived from the following:

(3) $$\left[\begin{array}{c} P_{xr}\\ P_{yr}\\ P_{zr} \end{array}\right]=\left[\begin{array}{c} x_{67}-y_{78}+z_{89}-y_{910}+l_{5}\\ y_{67}+x_{78}-y_{89}+z_{910}+l_{6}\\ z_{67}+z_{78}+x_{89}-x_{910}+l_{4} \end{array}\right]$$

Here, the distances between coordinate frames (e.g., $x_{01}$) are fixed by the mechanical assembly. Consequently, the end-effector’s position is governed solely by the displacement of each DOF (e.g., $l_{2}$). Therefore, once the end-effector’s position is identified by the microscope, the deflection from the desired position can be calculated. The deviation between the desired position and the current position of the manipulator is then used to compute the required displacement for each degree of freedom of the microrobot based on Equations (2) and (3). The resulting displacement is subsequently sent to the driver to generate the corresponding drive signals.

## 3. Shear Strength Characterization Method

### 3.1. Image Processing Method

During the measurement, the two ends of the test sample are fixed to the left and right manipulators, respectively. However, the assembling error may cause misalignment between the two ends, which can introduce errors in the direction and distribution of the applied shear force, ultimately reducing the accuracy of the measured shear strength. To ensure the shear force is applied along the intended direction, the sample must be properly aligned. This requires automated detection of the sample’s position, estimation of its current slope and mounting offset, and subsequent robotic adjustment to correct the misalignment. In this section, we use a copper wire sample (Shengshi Jingxin New Material Co., Ltd., Kunshan, China) to illustrate the image recognition and alignment process in detail.

The image of the copper wire is captured by a microscope (Dino, am73915mzt), which uses eight LED lights as its source of illumination. To achieve real-time image processing, we capture the image directly from the screen and then use Visual Studio to handle the image.

**Figure 3:**
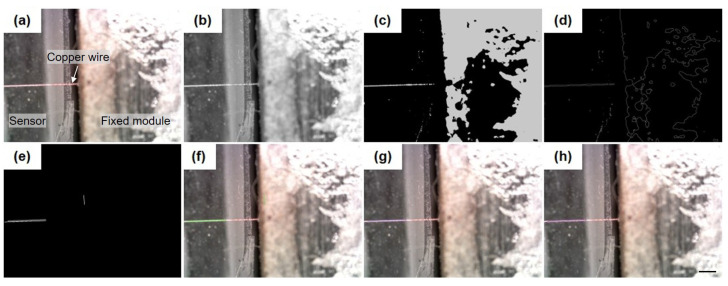
The image processing method for recognizing the copper wire: (**a**) original image, (**b**) gray image; (**c**) thresholding, (**d**) edge detection; (**e**) line detection, (**f**) contours; (**g**) selected contours; (**h**) middle line. Scale bar: 500 μm.

The image processing pipeline begins by converting the original image (Figure 3a) to grayscale and applying Gaussian filtering to reduce noise [28] (Figure 3b). The grayscale image is then thresholded to produce a binary image (Figure 3c), highlighting the key structural features of the sample. Edge detection is subsequently performed using the Canny operator [29], yielding the result in Figure 3d. Although the copper wire’s contour is clearly detected, it does not form a straight line, preventing direct estimation of its slope. To address this, the Hough transform is employed to detect straight-line segments within the image [30], as shown in Figure 3e. From the multiple detected segments, those corresponding to the copper wire are selected based on prior knowledge: since the wire’s inclination should be moderate, only lines with an angle below $45^{{}^{\circ}}$ are retained (Figure 3g). Finally, the slopes of the selected contours are averaged to estimate the overall orientation of the sample. The midline between the two contours is constructed, and its slope is taken as the representative slope for alignment evaluation. The result of this midline is illustrated in Figure 3h.

At this stage, the sample’s slope can be obtained in real time from image data. To correct the misalignment, the robot’s position is adjusted based on this slope: if the slope is positive, the left robot moves in the negative y-direction; otherwise, it moves in the positive y-direction.

### 3.2. Fabrication and Calibration of the Force Sensor

#### 3.2.1. Fabrication of the Force Sensor

Current methods for microscale force measurement often rely on detecting shape deformation in soft sensors. However, such approaches face two major limitations: the deformation may exceed the microscope’s field of view due to the sensor’s compliance and large strain, and the sensor structure must be carefully customized according to the sample being tested. To overcome these challenges, we develop a soft force sensor that generates a voltage signal in response to applied force.

The sensor consists of a piezoelectric PVDF film (MEAS, USA) encapsulated within a polydimethylsiloxane (PDMS) shell. The PVDF acts as a sensitive element that converts mechanical stress into an electrical output, while the PDMS shell easily shapes into various sizes and geometries via mold-based fabrication, which provides structural flexibility and protection. The following section details the sensor fabrication process. The PVDF film is first secured onto a 3D-printed mold. PDMS (SYLGRAD 184, DOWSIL, Midland, MI, USA) is then prepared by mixing the base and curing agent at a 10:1 weight ratio and stirring the mixture thoroughly for 10 min. Subsequently, the mixture is placed under vacuum for 1 h to remove air bubbles. The degassed PDMS is carefully poured into the mold and cured at 55 °C for 4 h. After demolding, the soft force sensor is obtained, with final dimensions of 65×22×5 mm.

#### 3.2.2. Calibration of the Force Sensor

For calibration, the force sensor is mounted on the microrobotic system, with a force meter (M3813A, Yuli, China) fixed directly above it. The charge from the sensor is amplified and transferred (by PVA 103, Weijingyi, China) to the voltage signal and observed by an oscilloscope (DS-1202, Rigol, China). During the calibration, the system moves in the z-direction to create contact between the sensor and the force meter. Throughout this process, both the contact force and the corresponding sensor voltage are recorded to establish a relationship between the two variables. We change the free length of the fixed sensor and the moving speed, and then measure the voltage and force in various situations. For each calibration condition, we conduct five experiments.

**Figure 4:**
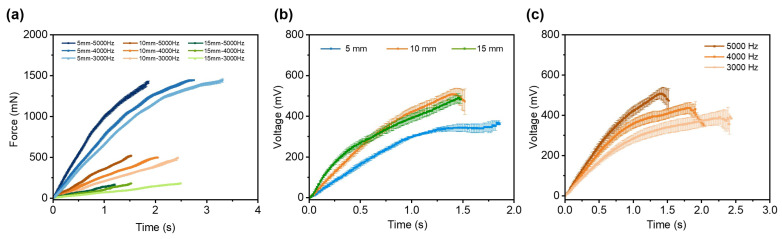
The shear force and measured voltage of the sensor change under different actuation frequencies and free lengths. (**a**) The shear force curve changes with time for various free lengths of the sensor. (**b**) The voltage curve changes with time under various free lengths of the sensor with a moving frequency of 5000 Hz. (**c**) The voltage changes with time under various moving frequencies at a free length of 10 mm of the sensor.

The calibration results reveal that the sensor’s response, especially its maximum output voltage and the slope of the force–voltage relationship, depends on both the sensor’s free length and the system’s actuation frequency (moving speed). Consequently, measurements are taken across a range of free lengths (5 mm to 15 mm) and actuation frequencies (3000 Hz to 5000 Hz). The results are presented in Figure 4.

Figure 4a displays the measured shear force profiles of the force sensor with different free lengths and actuation frequencies. The blue curve corresponds to a sensor with a 5 mm free length, while the orange and green curves represent free lengths of 10 mm and 15 mm, respectively. Variations in shading within each color indicate different actuation frequencies of the robotic system. The force exhibits an approximately linear increase over time during loading. For sensors of the same free length, the maximum measured force remains largely consistent. This is because, under identical deformation conditions, the force applied to a sensor is determined primarily by its free length. However, even when the free length is the same, the time required for the sensor to reach this maximum force varies. This difference arises because a lower actuation frequency results in a slower movement speed, leading to a gradual deformation process and consequently a shallower slope in the force–time curve. Furthermore, a shorter free length allows the sensor to withstand and accurately measure larger forces. This behavior is consistent with Euler–Bernoulli beam theory: under the same deflection (i.e., deformation displacement), the force experienced by the sensor is inversely proportional to the cube of its free length. Consequently, reducing the free length substantially enhances the structural stiffness, enabling the sensor to endure greater forces under equivalent deformation conditions. This highlights the critical role of free length in optimizing the force measurement range of soft sensors. We observe that the highest forces occur at the shortest free length (5 mm), with force amplitude decreasing as the free length increases to 15 mm. For any given free length, higher actuation frequencies produce greater peak forces. Conversely, lower frequencies yield slower movement, prolonging the time required to achieve maximum force. The corresponding voltage outputs are compared in Figure 4b,c. At a fixed actuation frequency of 5000 Hz (Figure 4b), the maximum voltage is observed at a free length of 10 mm (507.2 ± 25.67 mV), a value closely matched by the 15 mm length (490 ± 22.03 mV). Figure 4c shows the voltage response for a fixed free length of 10 mm across different frequencies. The highest frequency (5000 Hz) yields both the maximum voltage (507.2 mV) and the sharpest slope, indicating that a faster moving speed enables the sensor to measure larger forces with a higher resolution. This effect comes from the sensor’s high sensitivity to dynamic forces. Thus, increased driving frequencies induce more rapid force changes, which, in turn, produce more obvious voltage variations.

**Figure 5:**
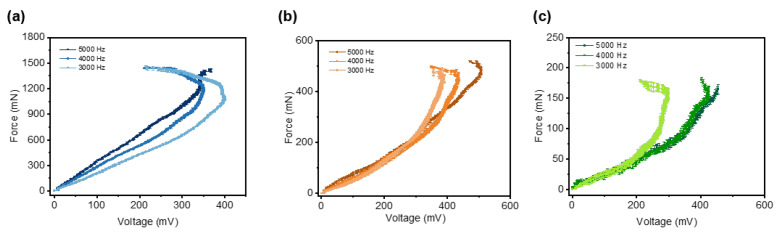
The relationship between measured force and voltage with different free lengths and actuation frequencies. (**a**) The relationship between force and voltage of the sensor with a free length of 5 mm. (**b**) The relationship between the force and voltage of the sensor with a free length of 10 mm. (**c**) The relationship between the force and voltage of the sensor with a free length of 15 mm.

Next, we present the relationship between the output voltage and the measured force under different actuation frequencies and free lengths in Figure 5. As shown in Figure 5a, the voltage–force relationship is approximately linear up to about 300 mV. Beyond this point, the response becomes nonlinear and eventually plateaus due to the dynamic sensitivity limit of the sensor. Furthermore, the slope at 5000 Hz is sharper than that at lower frequencies, and its inflection point occurs at a higher force, indicating both greater measurement sensitivity and a wider dynamic range. A similar trend is observed for free lengths of 10 mm (Figure 5b) and 15 mm (Figure 5c). The variation in slope among these curves comes from the dynamic response characteristics of the sensor. When the actuation frequency is higher, the resulting faster movement speed causes more rapid deformation of the sensor, thereby producing a sharper slope in the response curve. Based on these results, we select an actuation frequency of 5000 Hz for all subsequent experiments as it provides the highest sensitivity and largest measurement span across all free lengths.

**Figure 6:**
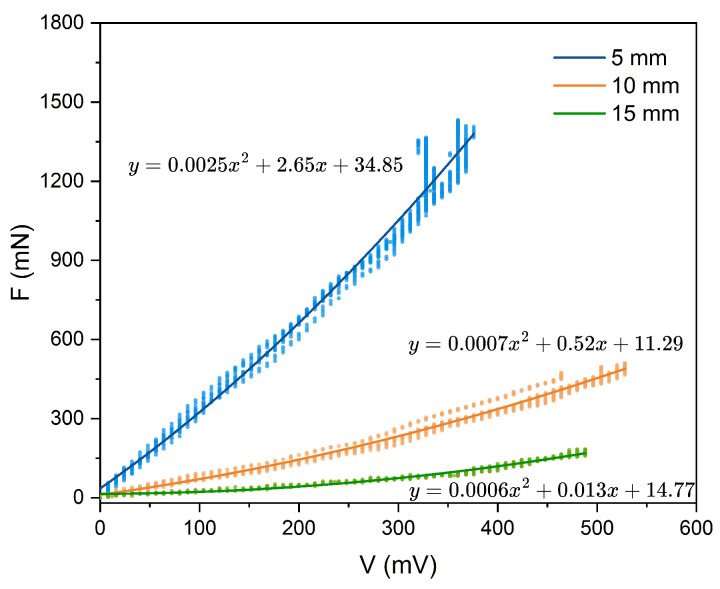
The fitting results of the voltage-force relationship for free length of 5 mm, 10 mm, and 15 mm.

We employ a second-order polynomial to model the relationship between voltage and force, with the fitting results presented in Figure 6. The $R^{2}$ values for the fits are 0.977, 0.983, and 0.980, respectively, indicating a strong model fit. The corresponding root mean square error (RMSE) values are 61.22, 18.44, and 6.87. During the shear force measurement experiments, the shear force acting on the sample will be determined based on the voltage signal acquired from the oscilloscope using the pre-established calibration relationship (fitting results) described above.

## 4. Experimental Results

### 4.1. Experimental Setup

**Figure 7:**
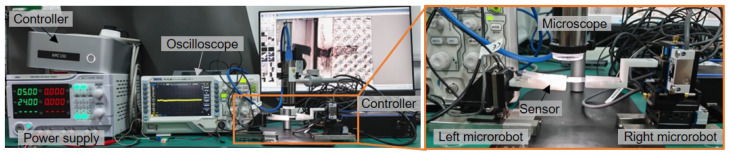
The experimental setup for the shear strength measurement.

The experimental setup for the shear strength characterization is shown in Figure 7. The experimental setup consists of the left microrobot (ECS3030, Attocube, Germany) with a soft sensor, the right microrobot (MN3-18, Mechonics, Germany) with a sample fixed stage, a microscope (Dino, am73915mzt), a power supply (A-BF, SS-305DM-2), and an oscilloscope (Rigol DS-1202). The soft force sensor is mounted on the left microrobot using a 3D-printed holder. The sample is fixed to a stainless steel sheet with adhesive tape (3M). Due to assembly tolerances, the initial position of the sample may not be properly aligned. Therefore, at the beginning of the measurement process, the microrobot moves within the X–Y plane to ensure the correct positioning of the sample. The robot then moves along the Z-axis to apply a shear force to the sample.

### 4.2. Experiment on Copper Wires

**Figure 8:**
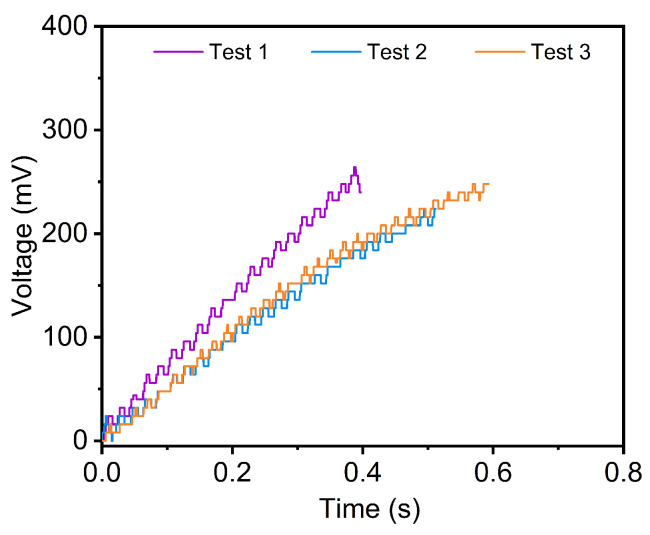
Each curve illustrates the temporal change in voltage output from the force sensor until fracture occurs. The consistent trends observed across all tests confirm the reliability of the measurement process. Notably, the slight variations between the curves are attributed to minor discrepancies in sample alignment, surface conditions of the wires, or differences in effective strain rates during loading.

**Figure 9:**
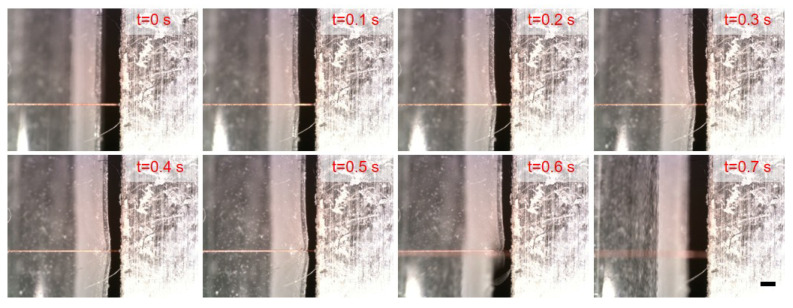
The experimental images of the shear process for measuring shear strength of copper wire. Scale bar: 200 μm. Between t = 0.1 s and 0.6 s, sensor deformation increases with applied force, followed by abrupt shear failure of the copper wire between t = 0.6 s and 0.7 s.

The shear strength of an oxygen-free copper wire is measured using a soft force sensor with a free length of 10 mm. The experimental results, presented in Figure 8, show consistent trends across all three test curves, indicating good repeatability of the measurement process. A representative sequence from Test 3 (Figure 9) illustrates the experiment process. After sample alignment, the sensor moves along the z-axis. Between t = 0.1 s and 0.6 s, sensor deformation increases with applied force, followed by abrupt shear failure of the copper wire between t = 0.6 s and 0.7 s.

The shear strength measurement results are summarized in Table 1. The measured shear strength values from the three tests are 107.7 MPa, 100.0 MPa, and 102.9 MPa, yielding a mean value of 103.5 MPa with a standard deviation of 3.9 MPa. The measured values align with typical shear strength ranges reported for oxygen-free copper (90–120 MPa) and experimental (108.230–124.973 MPa) and theoretical ranges (104.124–120.231 MPa) from [31,32,33,34,35,36], validating the experimental setup. The slight variation observed may be attributed to minor differences in sample alignment, wire surface conditions, or strain rate effects during testing. These results demonstrate good repeatability of the proposed method, given the challenges of soft sensor-based mechanical testing. The agreement between the measured and literature values supports the use of this soft sensor approach for reliable shear strength characterization in microscale specimens.

### 4.3. Experiments on Graphite Films

**Figure 10:**
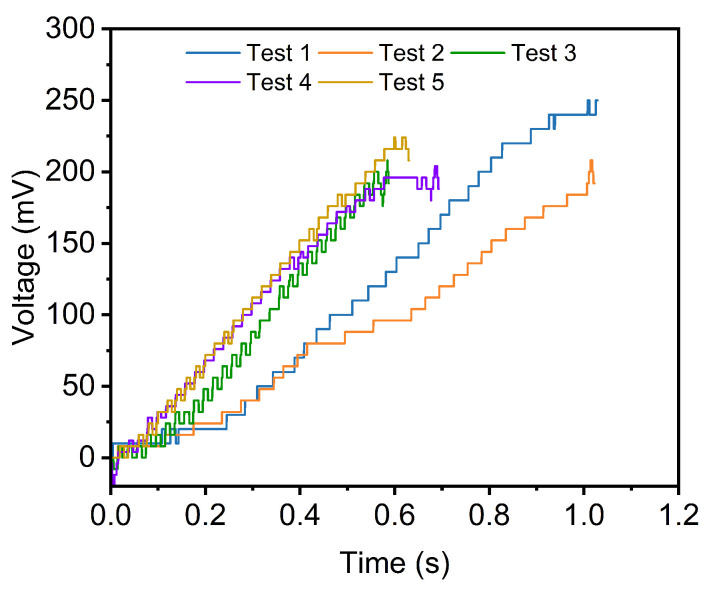
The changing curve of the voltage during shear strength measurement of graphite films.

**Figure 11:**
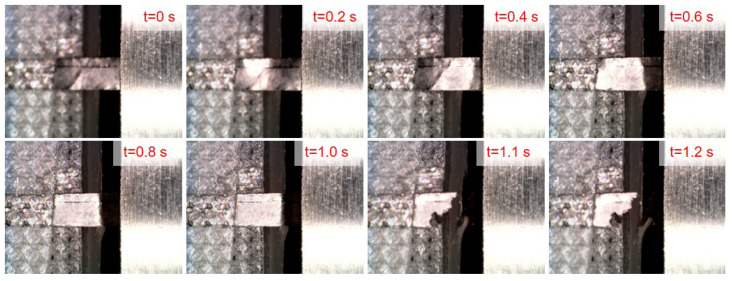
The shear strength measurement process for graphite film.

The shear strength of a 25 μm thick graphite film (Yige, China) is subsequently evaluated. The voltage–time profiles from five independent tests are presented in Figure 10, all of which exhibit consistent trends, thereby demonstrating the high repeatability of the proposed measurement method. A representative experimental sequence is illustrated in Figure 11. From t = 0 s to t = 1.0 s, the sensor moves along the z-axis, during which the displacement of the graphite film is visibly observed via reflection. Fracture of the graphite film occurs abruptly between t = 1.0 s and t = 1.1 s. The measured shear strength values from the five experiments are summarized in Table 2. Although the maximum voltage differs, the shear strength measurement result is very close due to the size variation. The results are 3.65 MPa, 3.69 MPa, 3.62 MPa, 3.56 MPa, and 3.51 MPa, yielding a mean shear strength of 3.61 MPa with a standard deviation of 0.067 MPa. The low standard deviation and coefficient of variation (1.86%) further confirm the high repeatability of the measurements. The close agreement between all five trials underscores the robustness of the methodology.

### 4.4. Discussion

Based on the experimental validation, the proposed microrobotic system demonstrates significant advantages in automated and high-precision shear strength characterization at the micro scale. It achieves full automation in sample alignment, force application, and calculation without manual intervention, while also exhibiting high repeatability across various sample geometries, as evidenced by low standard deviations in tests on copper wires and graphite films. However, there are some limitations of the current work. First, the current method focuses primarily on measuring shear strength. The measurement of other material properties, such as tensile strength or stiffness, requires redesigning the gripping mechanism and force sensor dimensions, along with recalibration. Second, due to the dynamic characteristics of the flexible sensor, higher force loading speeds are necessary to achieve a higher measurement accuracy and larger range. Hence, the measurement errors will increase if the loading speed is very slow. Furthermore, image processing parameters need to be adjusted based on the measurement target to achieve reliable object recognition. In the future, we can use deep learning or even large vision models to achieve more reliable and faster sample recognition, particularly for irregularly shaped or structurally complex samples that do not exhibit clear and regular contours. Moreover, by designing multi-axis force sensors and a universal gripping mechanism, we can expand the proposed system to multi-target measurement of diverse materials such as 2D structures and biological samples in multiple dimensions.

## 5. Conclusions

This paper presents an automated characterization approach based on a micro-robotic system, which bridges a critical gap in microscale mechanical testing. The main contributions of this work are as follows: (1) the development of a microrobotic system with 6-DOF for precise sample alignment and application of shear force; (2) the design of a machine vision algorithm for automatic sample identification and closed-loop robotic alignment; (3) the design and integration of a PVDF force sensor for real-time, in-situ shear force measurement with high sensitivity. By integrating image processing and a soft force sensor into the microrobotic system, the proposed method achieves automatic, high-precision, and excellent repeatability in measuring the shear strength of microscale materials. The measured shear strength values for oxygen-free copper wires align closely with established literature values, confirming the validity of the technique. The method is further validated by its application to ultra-thin graphite films, demonstrating its generalization ability across different material systems. Despite slight variations arising from assembly tolerances and material heterogeneity, the system overall shows strong potential for reliable micro-mechanical testing. This work offers a general, effective, and convenient automated solution for mechanical property characterization at the microscale. In the future, by developing various interchangeable fixing modules integrated with the microrobot, the system can be extended to support multi-axis, multi-dimensional, and multi-objective force measurements.

## Figures and Tables

**Table 1:** The shear strength measurement results for copper wires.

	Test 1	Test 2	Test 3
Maximum voltage (mV)	264	224	248
Shear force (mN)	199.7	164.7	185.4
Diameter (μm)	48.6	45.8	47.9
Shear strength (MPa)	107.7	100.0	102.9

**Table 2:** The shear strength measurement results of graphite films.

	Test 1	Test 2	Test 3	Test 4	Test 5
Maximum voltage (mV)	250	208	208	204	224
Shear force (mN)	187.2	151.3	151.3	148.0	164.7
Width (mm)	2.05	1.64	1.67	1.66	1.88
Shear strength (MPa)	3.65	3.69	3.62	3.56	3.51

## Data Availability

All data generated or analyzed during this study are included in this published article.
